# Bovine abortions due to the rare *Salmonella* serovars Kingston and Kedougou

**DOI:** 10.1177/10406387251324261

**Published:** 2025-02-28

**Authors:** Laurie Boucher, Pierre Hélie, Julie-Hélène Fairbrother, Sonia Chénier, Samuel Morin, Guillaume St-Jean

**Affiliations:** Departments of Veterinary Biomedical Sciences, Faculté de médecine vétérinaire, Université de Montréal, Saint-Hyacinthe, Québec, Canada; Pathology and Microbiology, Faculté de médecine vétérinaire, Université de Montréal, Saint-Hyacinthe, Québec, Canada; Laboratoire de santé animale de Saint-Hyacinthe, Ministère de l’Agriculture, des Pêcheries et de l’Alimentation du Québec (MAPAQ), Saint-Hyacinthe, Québec, Canada; Laboratoire de santé animale de Saint-Hyacinthe, Ministère de l’Agriculture, des Pêcheries et de l’Alimentation du Québec (MAPAQ), Saint-Hyacinthe, Québec, Canada; Bureau Vétérinaire Iberville, Saint-Jean-sur-Richelieu, Québec, Canada; Pathology and Microbiology, Faculté de médecine vétérinaire, Université de Montréal, Saint-Hyacinthe, Québec, Canada

**Keywords:** abortion, bovine, *Salmonella* Kedougou, *Salmonella* Kingston

## Abstract

Two aborted bovine fetuses were submitted (6 mo apart) with portions of their chorioallantois for postmortem examination. In both cases, microscopic examination revealed large numbers of gram-negative bacilli in the chorionic vessels and, to a lesser extent, in capillaries and venules of fetal organs of one fetus; the bacteria were not associated with noticeable inflammation or necrosis. *Salmonella enterica* subsp. *enterica* serovar Kingston was isolated from the placenta, abomasal contents, lung, and liver in case 1; *Salmonella* serovar Kedougou was isolated from the abomasal contents, lung, and liver in case 2. Both are rare serovars that have been isolated from various species but are not known to cause clinical disease in cattle, and, to our knowledge, have not been reported as the cause of abortion in dairy cattle.

*Salmonella* spp. are gram-negative, facultatively anaerobic rods of the *Enterobacteriaceae* family.^7^ *Salmonella* is a common pathogen in cattle, causing enterocolitis, septicemia, and abortion.^11^ *Salmonella* infections can also be subclinical, allowing transmission in livestock. Although *Salmonella* spp. have the potential to cause abortion, abortion is not a common outcome.^7^ There are many *Salmonella* serovars, some of which pose a zoonotic risk. Therefore, it is important to characterize the serovars causing clinical disease in animals to protect human and animal health.

In cattle, most isolated *Salmonella* spp. are from serogroups B–E. Serogroups B, C, and E are non–host specific; group D, represented by *Salmonella* Dublin, is host-adapted.^7^ *Salmonella* Dublin is of great concern in the bovine industry given its severe clinical manifestation associated with its host adaptability and invasion ability.^16^ Some of the most commonly isolated serovars in cattle are *Salmonella* Typhimurium (group B) and the host-adapted *Salmonella* Dublin (group D), although the prevalence of different serovars can vary greatly based on the geographic region as well as beef versus dairy production.^6,7^

*Salmonella* Kingston (group B) is a rare serovar isolated mainly from poultry and swine; *Salmonella* Kedougou (group G) is a serovar mainly associated with foodborne illness in humans in Europe and other continents.^5,8,15^ There is little if any evidence of disease in cattle in association with either serovar. Here we describe, from different farms, 2 bovine abortion cases due to *Salmonella* Kingston and Kedougou. We retrieved no cases of abortion in cattle associated with these serovars in a search of Google and PubMed using a mix of the different search terms ”Kingston”, “Kedougou”, “abortion”, “cattle”, suggesting that these serovars have not been reported in cattle, moreover with an associated disease condition.

An entire fetus (case 1), of 65-cm crown-rump length, was submitted with sections of the fetal membranes, including chorioallantois and cotyledons, for postmortem examination to the Centre de Diagnostic Vétérinaire de l’Université de Montréal (CDVUM) at the Faculté de médecine vétérinaire (Saint-Hyacinthe, Quebec, Canada). The fetus came from a Jersey dairy farm with 80 animals. This was the third abortion recorded in a 3-mo period in this herd. The cow was in its last trimester of gestation. There had also been episodes of diarrhea 2 wk before the abortion, but additional investigations had not been performed. There were no reported feed changes, nor any underlying disease of importance in the herd. It was reported that 50 animals had been imported from the United States 6 mo before this first abortion occurred. The animals were vaccinated against bovine alphaherpesvirus 1 (BoAHV1), bovine parainfluenza virus 3 (BPIV3), and bovine respiratory syncytial virus (BRSV) with a modified-live virus (MLV) vaccine and 5 serovars of *Leptospira interrogans* (Canicola, Grippotyphosa, Hardjo, Icterohaemorrhagiae, Pomona). They were not vaccinated against *Salmonella* spp.

An entire fetus (case 2), of 95-cm crown-rump length, was submitted with chorioallantois and cotyledons to our laboratory. The fetus was from a Holstein dairy farm with a herd of 150 animals. This was the second abortion in a 1-wk period in the herd. The cow was in its last trimester of gestation and aborted one month before its due date. The cows were vaccinated using a BoAHV1, BPIV3, BRSV (MLV), and a *Mannheimia haemolytica* toxoid commercial product, but not vaccinated against *Salmonella* spp. The veterinary clinician reported no additional clinical signs in the dam aside from the abortion. There was no recent change of nutrition in the herd.

The fetus in case 1 had moderate postmortem autolysis, and gross examination did not reveal any interpretable changes. Tissues were fixed in 10% neutral-buffered formalin, paraffin-embedded, cut at 3-μm thickness, and stained with hematoxylin–phloxine–saffron for microscopic evaluation. Histologically, the chorionic vessels of the chorioallantois were filled with gram-negative bacilli with no obvious associated inflammation or necrosis (Fig. 1A). Similar bacilli were also noted in capillaries and venules of the brain, heart, skeletal muscles, and small intestine (Fig. 1B, 1C). Squames and occasional amorphous eosinophilic material (possibly meconium) were present in the pulmonary alveoli, consistent with amniotic fluid aspiration. Gram-negative bacilli were found in a direct smear of the abomasal fluid. There were no other significant microscopic changes observed.

**Figure 1.**
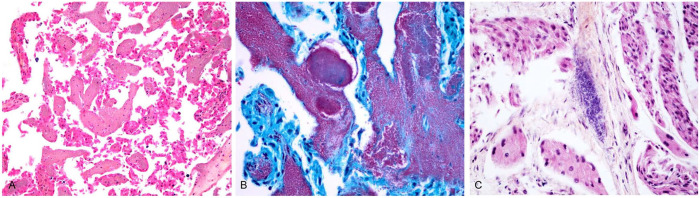
*Salmonella enterica* subsp. *enterica* serovar Kingston abortion in an aborted bovine fetus. **A.** Placenta with embolized bacteria within blood vessels and placental villi. Hematoxylin–phloxine–saffron stain (HPS). **B.** Gram-negative bacteria in the placenta (in red). Modified Gram Twort stain. **C.** Heart with embolized bacteria within a blood vessel. HPS.

The fetus in case 2 had moderate postmortem autolysis, and gross examination did not reveal any interpretable changes. On microscopic examination, the chorionic vessels of the chorioallantois, notably in the cotyledons, were filled with gram-negative bacilli with no obvious associated inflammation. There was multifocal necrosis of the chorionic epithelium (Fig. 2A). A direct smear of the abomasal fluid revealed gram-negative bacilli (Fig. 2B). There were no other microscopic changes observed.

**Figure 2.**
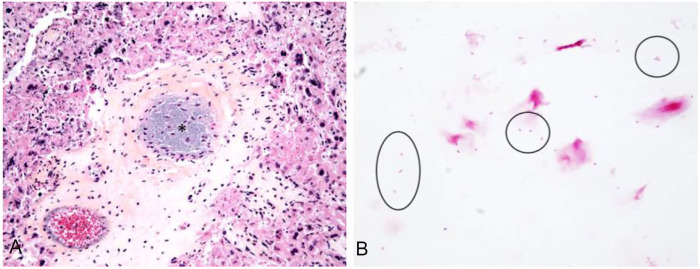
*Salmonella enterica* subsp. *enterica* serovar Kedougou abortion in an aborted bovine fetus. **A.** Placenta with embolized bacteria (*) within blood vessels and necrosis of chorionic epithelium. HPS. **B.** Direct smear of abomasal content with numerous gram-negative rods (circled). Gram stain.

A real-time PCR (rtPCR) panel was performed for *Neospora caninum*, BoAHV1, bovine viral diarrhea virus (BVDV), *Coxiella burnetii*, and *Leptospira* spp. on chorioallantois, brain, lungs, liver, spleen, and kidneys, and was negative in both cases.

Samples of placenta, abomasal contents, lung, and liver were submitted for routine bacterial culture. The samples were cultured on Columbia agar with 5% sheep blood at 35 ± 2°C with 5% CO_2_. In case 1, a heavy pure growth of group B *Salmonella* was isolated from most samples, with contaminants only in the placenta (although in lesser numbers than the *Salmonella*). Isolates were subjected to identification with matrix-assisted laser desorption/ionization time-of-flight mass spectrometry (MALDI-TOF MS; Bruker). Serotyping was done using the traditional agglutination assay. Because the Kingston serovar was detected for the first time in our laboratory, confirmation was done by the Public Health Agency of Canada (PHAC)–Laboratory for Foodborne Zoonoses (LFZ), Office International des Epizooties–Reference Laboratory for Salmonellosis, *Salmonella* Typing Laboratory (Guelph, Ontario, Canada). The complete antigenic formula is I:1,4,[5],12,[27]:g,s,t:[1,2]. The isolate from the liver was subjected to antimicrobial susceptibility testing by the Kirby–Bauer disk diffusion method according to the most recent guidelines published by the Clinical Laboratory Standards Institute (CLSI). When the CLSI criteria were unavailable for cattle or bacteria isolated from animals, the criteria for humans were used for the interpretation. The following antimicrobials were tested and reported: ampicillin, ceftiofur, enrofloxacin, florfenicol, sulfisoxazole, tetracycline, and trimethoprim–sulfamethoxazole. The strain was susceptible to all of these antimicrobials.

In case 2, pure moderate (lung) to heavy (liver, abomasal content) growth of group G2 *Salmonella* was found from all samples. Serovar Kedougou was identified and confirmed at the LFZ. The complete antigenic formula is I:1,13,23:i:l,w. By Kirby–Bauer disk diffusion, the strain was susceptible to all tested antimicrobials mentioned above except for sulfisoxazole, which was interpreted as non-susceptible.

Our final diagnosis was abortion caused by *Salmonella enterica* subsp. *enterica* serovar Kingston in case 1 and *Salmonella* Kedougou in case 2. Causes of abortion in cattle can be either noninfectious (e.g., idiopathic, genetic, nutritional) or infectious (e.g., *Neospora caninum*, BoAHV1, *Leptospira* spp., BVDV). *Salmonella* spp. are known to cause abortion in the last trimester, as observed in our cases, which is a critical period because the fetus has a very rapid growth rate at this time. However, *Salmonella* abortion remains uncommon in dairy cattle.^9,11,17^ *Salmonella* Kingston and Kedougou are rare serovars that are not known bovine pathogens. We found no reports of abortions in domestic (feline, canine, etc.) or production (bovine, caprine, etc.) animals associated with these serovars. However, in a 2013 study, serovar Kingston was isolated from dead guinea fowl embryos, demonstrating its potential to infect fetuses of other species.^2^ Although the occurrence of recent abortions in both herds may suggest a predominant role for these *Salmonella* spp., the lack of additional investigations in the other aborted fetuses and the cows prevents us from confirming this hypothesis. Furthermore, the rare occurrence of these serovars in the literature (and cattle) could suggest isolated cases.

*Salmonella* spp. are usually transmitted via fecal–oral contamination and can cause a wide range of clinical signs in cows, varying from enterocolitis to pneumonia, septicemia, and abortion. Difficulties in controlling salmonellosis arise from the presence of potential subclinical cattle (carriers) that can spread the bacteria in the herd’s environment.^7^ The development of *Salmonella*-related disease is often associated with underlying risk factors, mostly stress-related factors inducing decreased immune responsiveness. These factors include, notably, the individual immune status of each animal (including an increased risk in younger animals), concomitant disease (viral or bacterial), the load of bacteria in the environment (often increased by undetected carriers), and environmental factors, such as variations in temperature, lack of biosecurity measures, and poor hygiene.^16^

*Salmonella* spp. are known to translocate into the intestinal lymphoid tissues and eventually into the bloodstream, causing bacteremia or sepsis. During pregnancy, the bacterium can then implant in the uterus and fetus, causing abortion, which we suspect to be the pathogenesis in our cases.^11,18^ The confirmation of bacterial abortion can be challenging, as careful interpretation of both the observed lesions and bacteriology results is mandatory to differentiate an etiology from a potential contaminant. In our case, the pure growth of *Salmonella* spp. noted in most of the submitted samples, including the stomach content, which is representative of the amniotic environment, and the emboli in multiple organs were sufficient to confirm the diagnosis. The lack of inflammation associated with the emboli in case 1 is a feature occasionally reported in acute bacterial abortion in cows.^1^ We suspect that the pathogenesis remains the same given the lack of inflammation, although we could not exclude that minimal or mild inflammation was masked by autolysis, or that other factors, such as the dam’s health status (fever, etc.), could have contributed to the pathogenesis.

*Salmonella* Kingston was isolated from fecal samples of guinea fowl in Benin, and *Salmonella* Kedougou was isolated in the feces of subclinical cattle in Algeria.^2,10^ Fecal isolates are expected, given that *Salmonella* spp. are mainly transmitted by a fecal–oral pathway.^7^ The diarrhea experienced in the first herd 2 wk before the abortion may have been attributed to the same bacterium, although this cannot be proven. It also seems improbable, as 4 PCR assays, 3 on feces and 1 in the reservoir, performed in the last year were negative for *Salmonella* spp. However, we can neither confirm nor deny that an infection occurred between the last surveillance PCR assay and the time of abortion. In an in vitro study, the potential of *Salmonella* Kingston to form strong biofilms was demonstrated, a feature that allows *Salmonella* Kingston to survive well in the environment.^19^ Because *Salmonella* Kingston may have contaminated the farm and may be circulating among animals, it would be of importance to continue the surveillance of *Salmonella* spp. in the herd and identify potential shedding.

*Salmonella* Kedougou is not as rare as *Salmonella* Kingston. There are many epidemic surveillance programs in Europe for various *Salmonella* serovars, including Kedougou.^15^ Moreover, an outbreak caused by this serovar was linked to contamination of salami in Norway in 2006 and of infant formula in Spain in 2008.^4,14^ *Salmonella* Kedougou thus appears to be associated with foodborne illness in humans, and has also been shown to be an important serovar causing human infections in northern Thailand.^12^ Although *Salmonella* Kedougou is important in human health, there are few if any cases of disease caused in cattle by this serovar, highlighting our interest in reporting bovine abortion caused by *Salmonella* Kedougou.

It is not possible to clearly understand how these 2 serovars were introduced into the environment of these farms, as they appear to be the first cases reported in cattle in Canada. We did note that some animals were imported from the United States 6 mo before case 1 occurred, although no conclusion could be drawn about whether the animals could have been carriers prior to their introduction in Canada, and no further investigations were performed to investigate the source herd. Interestingly, pigeons, which are known to be important vectors of *Salmonella*, had access to the cattle feeders in case 2. When this situation was corrected, the abortions stopped. Because *Salmonella* Kedougou was isolated from pigeon feces in a 2013 study,^4^ we could hypothesize that the pigeons were the source of infection in case 2^3^; however, no samples were taken from the cattle feeders. The PHAC reported 3 cases in humans of *Salmonella* Kedougou in 2019, 3 in 2022, and 1 in 2020, as well as 1 case of *Salmonella* Kingston through the National Enteric Disease Surveillance Program, demonstrating the presence of these serovars in Canada.^13^
